# Linkage, Evaluation and Analysis of National Electronic Healthcare Data: Application to Providing Enhanced Blood-Stream Infection Surveillance in Paediatric Intensive Care

**DOI:** 10.1371/journal.pone.0085278

**Published:** 2013-12-20

**Authors:** Katie Harron, Harvey Goldstein, Angie Wade, Berit Muller-Pebody, Roger Parslow, Ruth Gilbert

**Affiliations:** 1 Institute of Child Health, University College London, London, United Kingdom; 2 Graduate School of Education, University of Bristol, Bristol, United Kingdom; 3 Healthcare Associated Infection and Antimicrobial Resistance Department, Public Health England, London, United Kingdom; 4 Division of Epidemiology, University of Leeds, Leeds, United Kingdom; University of Cambridge, United Kingdom

## Abstract

**Background:**

Linkage of risk-factor data for blood-stream infection (BSI) in paediatric intensive care (PICU) with bacteraemia surveillance data to monitor risk-adjusted infection rates in PICU is complicated by a lack of unique identifiers and under-ascertainment in the national surveillance system. We linked, evaluated and performed preliminary analyses on these data to provide a practical guide on the steps required to handle linkage of such complex data sources.

**Methods:**

Data on PICU admissions in England and Wales for 2003-2010 were extracted from the Paediatric Intensive Care Audit Network. Records of all positive isolates from blood cultures taken for children <16 years and captured by the national voluntary laboratory surveillance system for 2003-2010 were extracted from the Public Health England database, LabBase2. “Gold-standard” datasets with unique identifiers were obtained directly from three laboratories, containing microbiology reports that were eligible for submission to LabBase2 (defined as “clinically significant” by laboratory microbiologists). Reports in the gold-standard datasets were compared to those in LabBase2 to estimate ascertainment in LabBase2. Linkage evaluated by comparing results from two classification methods (highest-weight classification of match weights and prior-informed imputation using match probabilities) with linked records in the gold-standard data. BSI rate was estimated as the proportion of admissions associated with at least one BSI.

**Results:**

Reporting gaps were identified in 548/2596 lab-months of LabBase2. Ascertainment of clinically significant BSI in the remaining months was approximately 80-95%. Prior-informed imputation provided the least biased estimate of BSI rate (5.8% of admissions). Adjusting for ascertainment, the estimated BSI rate was 6.1-7.3%.

**Conclusion:**

Linkage of PICU admission data with national BSI surveillance provides the opportunity for enhanced surveillance but analyses based on these data need to take account of biases due to ascertainment and linkage error. This study provides a generalisable guide for linkage, evaluation and analysis of complex electronic healthcare data.

## Introduction

Blood-stream infection (BSI) is an important cause of mortality, morbidity and substantial extra cost for paediatric patients, and paediatric intensive care units (PICU) have one of the highest rates of BSI of all specialties[1–4]. The national laboratory surveillance system coordinated by Public Health England (PHE, formerly the Health Protection Agency) collects data on microorganisms submitted by hospital laboratories in England and Wales[5]. Patient-level data on all children admitted to paediatric intensive care units (PICU) in England and Wales have been collected by the Paediatric Intensive Care Audit Network (PICANet) since 2003[6]. To date, no evaluation of the potential of linking these administrative data sources for national monitoring of risk-adjusted BSI trends in PICU has been performed[7,8].

There are two main obstacles to linkage for enhanced BSI surveillance. Firstly, as a voluntary system, PHE’s surveillance database (LabBase2) does not capture complete BSI data from all laboratories[5]. Hospital laboratories are requested to report any clinically significant bacterial infections and clinically significant isolates from sterile sites such as blood, although there are no specific guidelines for judgement of clinical significance and non-clinically significant isolates or contaminants may also be present in the data. Data are not always captured consistently, with staffing issues and IT compatibility problems causing incomplete and variable reporting over time. Ascertainment of MRSA and MSSA within LabBase2 in 2008 was estimated at around 70% (based on mandatory reports for methicillin-resistant and methicillin-susceptible *Staphylococcus aureus*) although ascertainment for all-cause bacteraemia in children is unknown[9].

Secondly, linkage between data sources is complicated due to a lack of well-completed unique identifiers in LabBase2. For data such as these, the method of choice for linkage is often to calculate probabilistic match weights (or match probabilities) that measure the similarity between records from different sources, taking into account possible identifier errors or missing values[10,11]. These weights or probabilities are then used to classify record pairs as links or non-links. 

Classification is typically based on highest-weighted (HW) pairs, where the candidate record with the highest weight is accepted as a link, given it exceeds a pre-specified threshold. However, errors can be introduced if the highest-weighted record is not the correct match (false-matches), or if no candidate record exceeds the threshold (missed-matches). An alternative classification method is prior-informed imputation, which aims to avoid bias associated with these linkage errors. Prior-informed imputation works by accepting values for variables of interest within a multiple imputation framework, rather than by linking a complete record[12]. Values are selected according to Information from a prior distribution (based on match probabilities in candidate linking records) combined with a likelihood derived from unequivocally-linked records[12].

There is a lack of practical guidance on the complex process required to link and analyse national administrative data such as PICANet and LabBase2. Methods used for data pre-processing, calculation of match weights or probabilities and errors due to mis-classification in the linkage process can have substantial effects on outcome measures[13–16]. We aim to describe the steps involved in preparing and linking routine data for enhanced BSI surveillance in PICU, which are generalisable to other administrative data of this type.

## Methods

### Ethics Statement

For PICANet, collection of personally identifiable data has been approved by the National Information Governance Board (Formerly the Patient Information Advisory Group) http://www.nigb.nhs.uk/s251/registerapp and ethical approval granted by the Trent Medical Research Ethics Committee, ref. 05/MRE04/17. PICANet also has specific permission from the National Research Ethics Service for linkage with the PHE laboratory data on bloodstream infections using personal identifiers and to share PICANet data with PHE. An exemption under Section 251 of the NHS Act 2006 (previously Section 60 of the Health and Social Care Act 2001) allows PHE to receive patient-identifiable data from other organisations without patient consent in order to monitor infectious disease. Specific permission for the PICANet-PHE linkage has been granted by NIGB. Consent for the use of the data identifying individual PICUs in this study was obtained by the relevant PICANet unit leads. Data in PICANet and LabBase2 cannot be publicly deposited as it is personally-identifiable. Access to an anonymised form of the linked data may be requested from http://www.picanet.org.uk/.

### Linkage process

**Figure 1:**
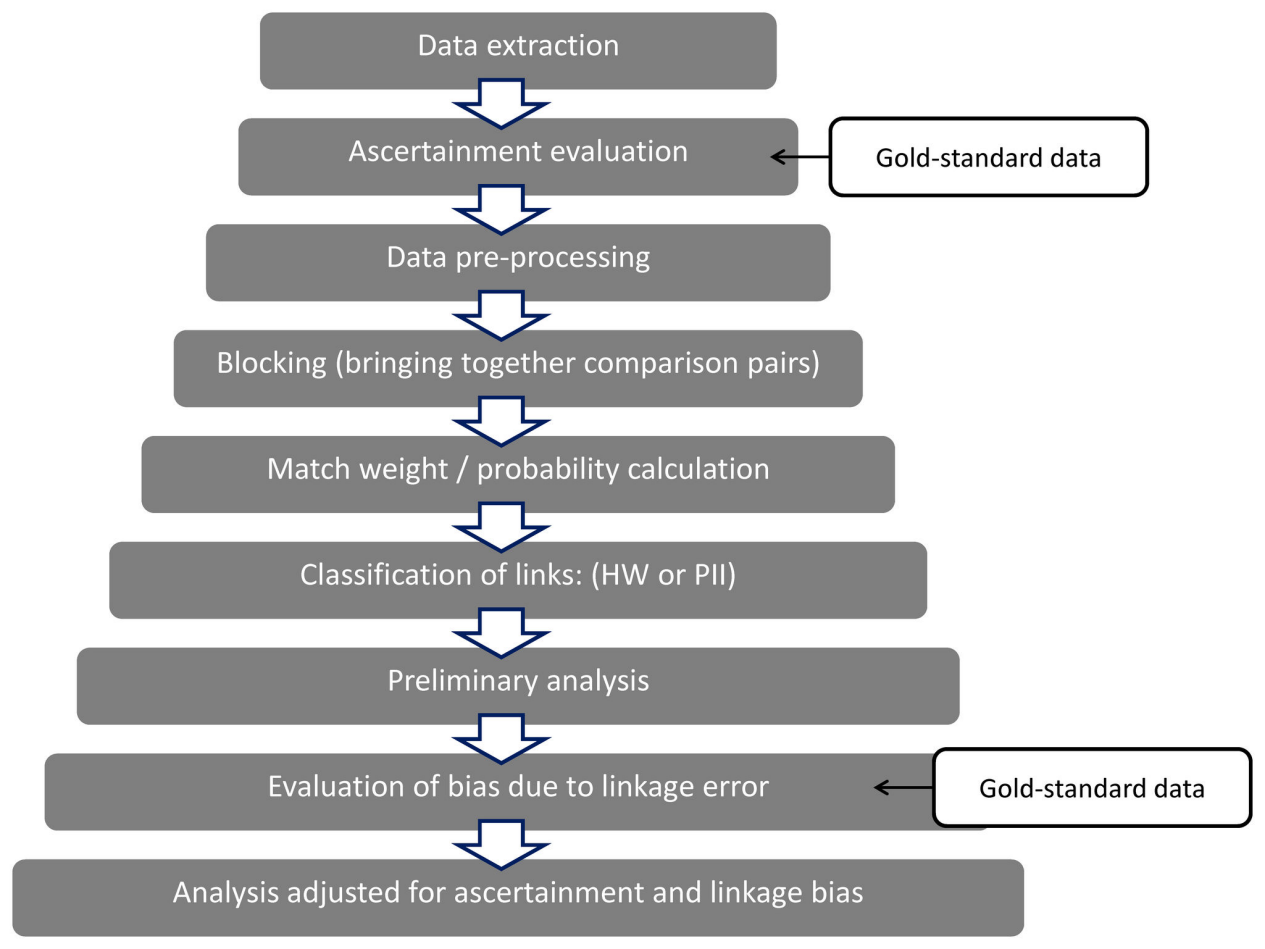
Steps involved in linkage of PICANet and LabBase2 for enhanced BSI surveillance in PICU.

Figure 1 displays the steps required for obtaining data for enhanced BSI surveillance through linkage between PICANet and LabBase2.

### Data extraction and de-duplication

Data on all PICU admissions for children <16 years for 2003-2010 were extracted from PICANet (n=109,654 records). Each PICANet record corresponded to an individual PICU admission within one of twenty-two PICUs admitting more than 200 children per year in England and Wales. Records of all positive isolates from blood culture captured by the national surveillance system were extracted for children <16 years between 2003-2010 extracted from LabBase2 (n=80,009).

PICANet admission records could link to none, one or more LabBase2 specimen records. If an admission record linked to multiple specimens of the same organism (within 14 days), only the first specimen was retained. LabBase2 specimen records could link to more than one admission if a specimen fell within the timeframe for two admissions at once (if the admissions were consecutive). In this case, the specimen was linked to the earlier of the two admissions, so that each LabBase2 record linked to at most one admission record. 

### Gold-standard data

“Gold-standard” microbiology datasets were obtained directly from three hospital laboratories: Birmingham Children’s Hospital (BCH), Oxford University Hospital (OUH) and Royal London Hospital (RLH). These laboratories were chosen as they were able to provide microbiology data that were eligible for reporting to LabBase2 (i.e. defined as clinically significant my laboratory microbiologists) and included unique identifiers. The gold-standard datasets were used for two purposes:

1. To estimate ascertainment in LabBase2 by comparing records that were eligible for reporting to LabBase2 with records that actually appeared in LabBase2 (BCH and RLH)
2. To evaluate linkage error by comparing records linked using incomplete identifiers within LabBase2 with records linked using well-completed unique identifiers within the gold-standard data (BCH and OUH). Any uncertain links in the gold-standard data were verified with additional information from the hospital.

The representativeness of the gold-standard data was assessed by comparing characteristics of laboratories and PICUs providing gold-standard data with those that did not.

### Ascertainment evaluation

Incomplete reporting in LabBase2 was identified through manual inspection of plots of the total number of reports (all ages) of bacteraemia for individual laboratories by specimen month. Data were inspected for all ages, as numbers for children only were low, and reporting gaps were expected to relate to the laboratory as a whole rather than to an individual ward. Within individual laboratories, months during which no reports were submitted were defined as a reporting gap. In addition, months during which an unrealistically small number of reports were present were defined as having incomplete reporting. Unrealistic numbers of reports were identified through careful manual inspection of reports over time: due to the fluctuation of reports from month to month and variation in size of laboratories, a consistent definition of incomplete reporting could not be applied across all laboratories and so a conservative judgement on incomplete reporting was made. Lab-months classified as incomplete reporting were excluded from analysis. 

For the remaining months, ascertainment of clinically significant BSI for children <16 years was estimated as the proportion of eligible records in the gold-standard data from BCH (March 2003-December 2010) and RLH (July 2006-December 2009) captured within LabBase2. BCH and RLH contained information on whether a specimen was deemed to be clinically significant (and therefore eligible for submission to LabBase2); this information was not available for OUH. 

### Data pre-processing

**Figure 2:**
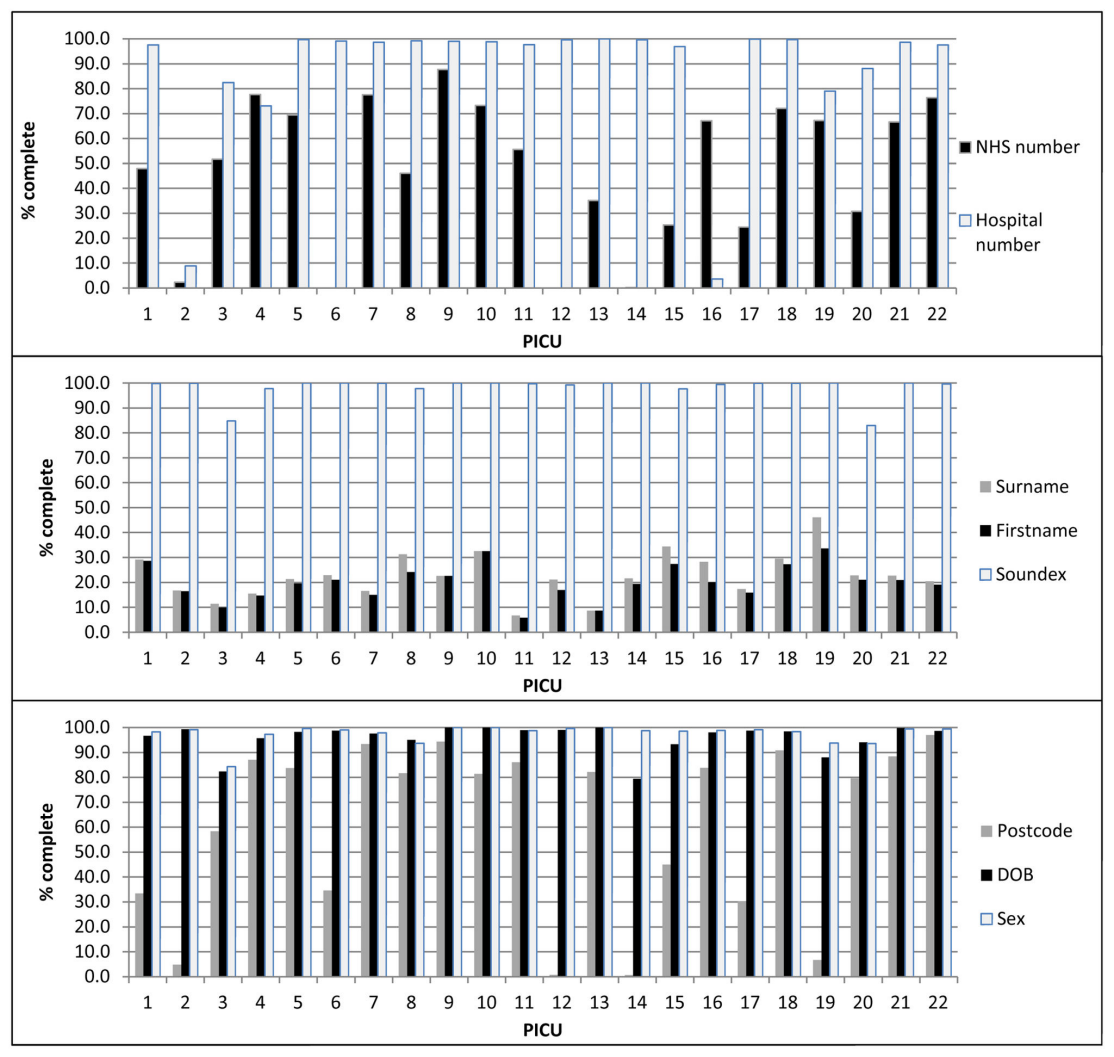
Completeness of identifiers in LabBase2 by PICU.

Completeness of common identifiers for linking varied between datasets and by time (identifiers were more complete in recent years). For LabBase2, completeness of identifiers varied by unit (Figure 2). For PICANet, date of birth and hospital number were 100% complete and the majority of other identifiers were >98% complete, with the exception of NHS number (85% complete). For both datasets, cleaning and data preparation were undertaken: NHS or hospital numbers such as “Unknown” or “9999999999” were set to null; generic names (e.g. “Baby”, “Twin 1”, “Infant Of”) were set to null; multiple variables were created for multiple surname and first names; postcodes beginning “ZZ” (indicating no UK postcode) were set to null. 

### Blocking

The total number of pairwise comparisons between PICANet and LabBase2 would produce of 80,009 x 109,654 = 8,773,306,886 comparison pairs. Comparison pairs were therefore restricted to those where the specimen date fell within 3 days of a PICU admission, as errors in date variables were assumed to be trivial (unpublished analysis showed <1% of specimen dates were inconsistent with data upload dates). To further reduce the number of comparison pairs, several blocking variables were chosen, so that records were only compared if they agreed on at least one of Soundex, initial, postcode prefix, NHS number, hospital number or day of birth. This blocking scheme assumed that records not agreeing on any of these blocking variables did not belong to the same individual.

### Match weight calculation

Match weight calculations were based on the Fellegi-Sunter method[11,17]. ‘Training’ datasets of record pairs assumed to be matches were used to estimate m- and u-probabilities P(agreement|match) and P(agreement|non-match) for individual identifiers. The first training dataset took records agreeing on NHS number or hospital number as assumed matches. The same training dataset was used to create a list of non-matches by cross-joining all record pairs and removing those agreeing on NHS number or hospital number. Frequency-based weights were calculated for surname, first name, sex and Soundex so that m- and u-probabilities were allowed to vary according to how rare or common a value was[18]. These were calculated by estimating m- and u-probabilities within groups for each identifier. For example, surnames beginning with Z were less common than surnames beginning with S, and the frequency-based weight represented this difference.

**Figure 3:**
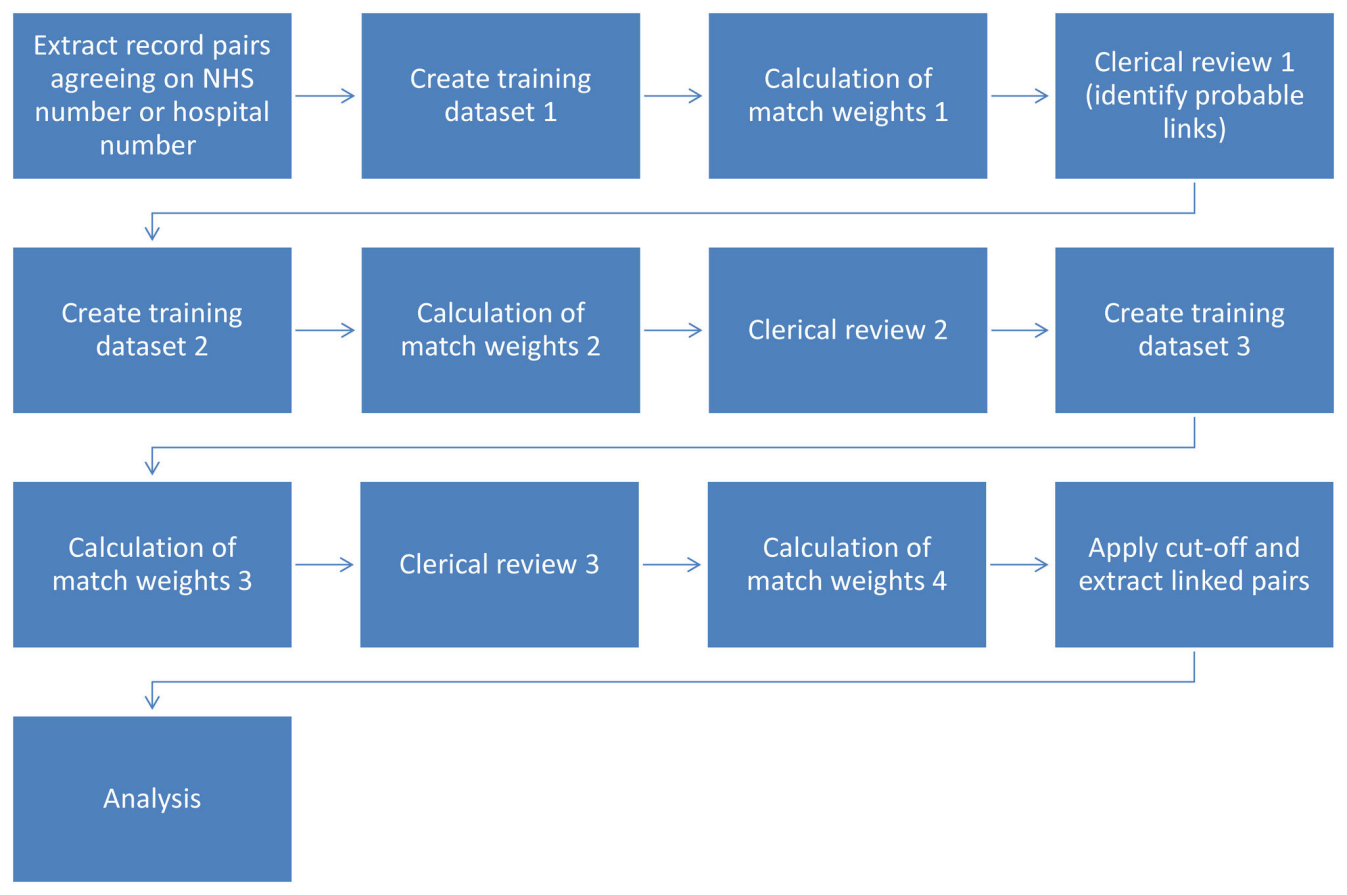
Match weight calculation process.

The distribution of match weights for links and non-links was plotted to assess the performance of the match weights at separating links and non-links. Record pairs were then ordered by match weight and manually inspected to identify obvious non-links that had high weights, and probable links with low weights. Subsequent training datasets were obtained by retaining probable links identified through this review. This process was iterated a number of times, until match weights from consecutive training datasets were stable (Figure 3).

### Match probability calculation

Match probabilities P(M|agreement pattern) were calculated to estimate the probability of a match given agreement on a joint set of identifiers. This avoided the assumption of independence between identifiers. Probabilities were derived as the number of links divided by the total number of pairs for each agreement pattern (based on probable links identified in the training datasets). For example, if 378 comparison pairs agreed on date of birth and Soundex but disagreed on sex, and 312 of these were probable links, the match probability for the agreement pattern [ 1,1,0 ] was 312/378=0.825.

### Classification of links

#### 1: Highest-weight (HW) classification

Traditionally, candidate linking records are ordered by match weight, and only the comparison pair with the highest probabilistic weight is classified as a link. All remaining candidate records are discarded (highest-weight classification). Comparison pairs are classified into non-links, links and uncertain links, based on the value of the match weight. Uncertain links are then classified through manually inspecting the identifiers on each record, to determine whether or not they belong to the same individual. Manual inspection makes use of the fact that the human eye can recognise matches that a computer would discard (e.g. Liz and Elizabeth) and can involve the use of additional identifiers if available. 

For this project, manual review for uncertain links was not possible. This was because no additional external data was available, and uncertain links often contained only Soundex and date of birth, which did not provide enough information to positively determine link status by eye. Records were therefore classified as links or non-links based on a single cut-off weight, based on capturing probable links identified in the training datasets. A sensitivity analysis was performed by repeating this process with two different cut-offs. The first cut-off (relaxed threshold) aimed to capture as many of the probable links as possible. The second cut-off (conservative threshold) aimed to exclude as many non-links as possible. Any records with a match weight above the threshold was classified as a link, and all others were classified as non-links.

#### 2: Prior-informed imputation)

Prior-informed imputation was performed as proposed by Goldstein et al, using Stat-JR software developed by the University of Bristol[12,19]. Linkage between PICANet and LabBase2 was ‘incomplete’, as PICANet records that did not have a BSI genuinely had no matching record in LabBase2. This is a special case for prior-informed imputation, as all candidate records had the same outcome (link = BSI).

**Figure 4:**
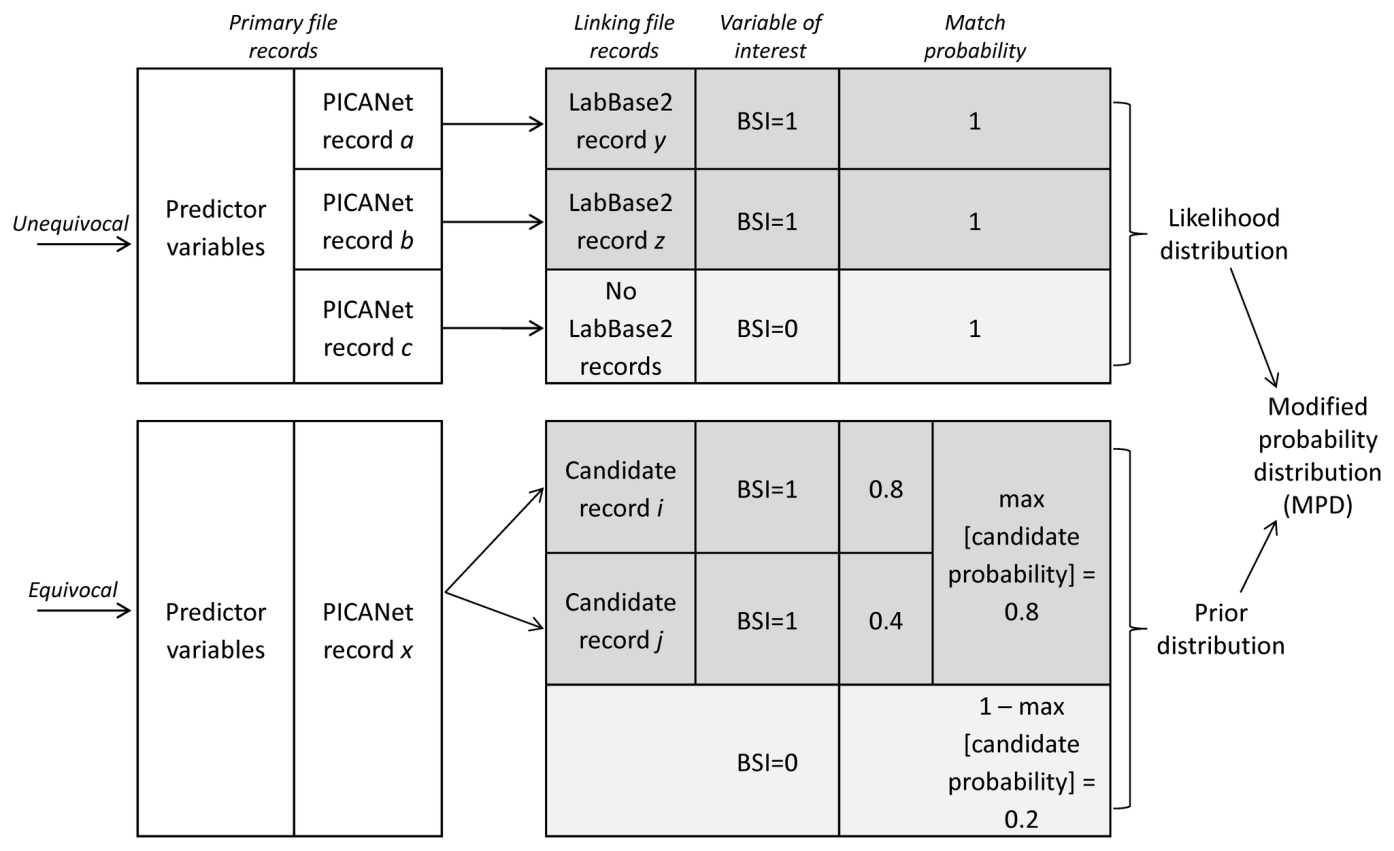
Prior-informed imputation for ‘incomplete’ linkage between PICANet and LabBase2. Predictor variables: Length of stay, age, admission type, admission source, renal status, quarter-year at admission.

Prior-informed imputation uses match probabilities, rather than match weights, to avoid assuming independence between identifiers. If a PICANet record had a match probability>0.9, it was classed as “unequivocal” and the variable BSI was set equal to 1 (Figure 4). If a PICANet record had no candidate linking records (or the maximum candidate probability was <0.1), it was also classed as “unequivocal”, but BSI was set equal to 0. These cut-offs were based on previous simulation work for prior-informed imputation. A likelihood for BSI was derived using the unequivocally linked records and a set of PICANet predictor variables identified in previous analyses[7].

For the remaining (equivocal) PICANet records, a prior distribution for BSI was created based on the maximum probability of a BSI in the candidate records (Figure 4). If the maximum candidate probability for BSI=1 if *p*, the probability that BSI=0 is 1-*p.*

A modified (posterior) probability distribution was created by multiplying the above prior distribution by the likelihood and scaling to 1. For each equivocal PICANet record, BSI was set equal to 0 or 1 according to the highest modified probability for that record. If no probability exceeded 0.1, BSI was treated as missing and standard multiple imputation was used to impute a value based on the likelihood only. Five imputed datasets were produced and analysed separately, with results combined using Rubin’s rules[20].

### BSI rate: Evaluation of bias due to linkage error

BSI rate was calculated as the percentage of admissions linked to >=1 BSI (within three days either side of admission). This crude rate reflects the proportion of admissions associated with an infection acquired either leading up to or during an admission. 

There are a number of ways in which bias due to linkage error can be evaluated[21]. Firstly, comparisons with gold-standard data can be performed, based on the true match status of any record pair (known in the gold-standard data). Such data could be in the form of an external dataset including well-completed, unique identifiers, or a sample of records that have been subjected to extensive manual review. In our study, bias was estimated by comparing the BSI rate in gold-standard data obtained directly from two laboratories, with that estimated in the linked data, for each classification method.

Secondly, sensitivity analyses based on varying linkage criteria can be used to provide a range of plausible results. This is particularly useful when aspects of the linkage process are subjective – for example, manual review or choice of thresholds. In our study, we present results based on two different probabilistic thresholds.

Thirdly, comparisons of linked and unlinked data can be made, in order to identify potential sources of bias. This process can help to identify groups of subjects who may be missed from the linkage due to poor data quality. In our study, differences in the characteristics of linked and unlinked records were related to BSI: linked records corresponded to admissions with BSI and therefore represented children who had risk-factors for BSI.

Finally, statistical techniques can be used to handle uncertainty in linkage within the analysis itself. In our study, we used prior-informed imputation to account for linkage error within analysis. 

## Results

### Ascertainment

**Figure 5:**
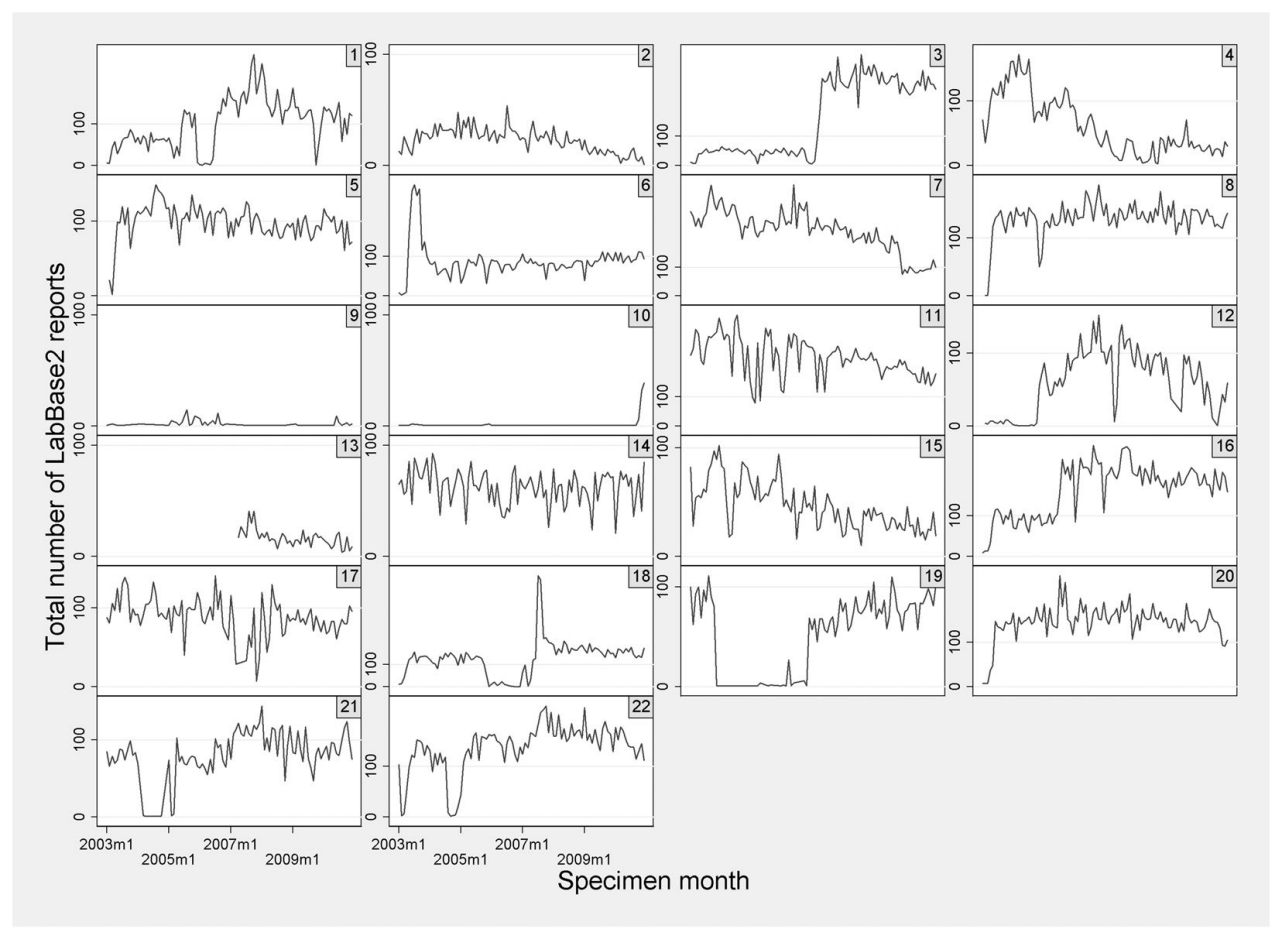
Total number of reports (all ages) submitted to LabBase2 for laboratories serving PICUs between 2003-2010.

Figure 5 shows the variation and fluctuation in the total number of reports (all ages) submitted to LabBase2 per month for laboratories serving individual PICUs between 2003-2010. Manual inspection of data identified a total of 548/2596 lab-months with incomplete reporting. Two laboratories had incomplete reporting for the entire study period (PICUs 9 and 10, Figure 5). Removing admissions during periods of incomplete reporting reduced the total number of admissions available for analysis from 109,654 to 78,525. Comparing the number of BSI records within BCH and RLH gold-standard datasets (defined as clinically significant by microbiologists) with BSI records captured by LabBase2 gave an estimated LabBase2 ascertainment (for clinically significant BSI in children <16 years) of 81.5% (95% confidence interval (CI) 79.9-83.1%; 1872/2298) and 79.5% (95% CI 75.1-83.9%; 260/327) for BCH and RLH respectively. As non-clinically significant specimens were also available in the gold-standard data, we identified that 181/2054 (8.8%) of LabBase2 reports for BCH were not clinically significant. 

### Blocking

After removing record pairs that were not within the correct timeframe (more than 3 days outside admission), there were 3,081,719 record pairs to be compared. Blocking on NHS number, hospital number, day of birth, Soundex, initial and postcode prefix, provided a total of 1,803,808 comparison pairs.

### Match weight and probability calculation

An initial three iterations of probabilistic weight calculation were performed. However, match weights did not stabilise. This was down to the failure of a number of assumptions underpinning probabilistic weight calculation. Firstly, all records should be equally likely to link. A small subset of LabBase2 records that did have well-completed data caused this assumption to fail, and weight calculations were dominated by agreement on NHS number, hospital number or name, making it difficult to distinguish between records containing only Soundex, date of birth and sex (Table 1). Secondly, agreement between identifiers should be independent. However, records with missing NHS number were also more likely to have missing surname, meaning that records failing to agree on NHS number were also likely to fail to agree on surname, meaning the independence assumption failed. The same was the case for Soundex and surname, and for separate elements of date of birth (records that disagreed on date of birth were disproportionately penalized). Finally, some weights produced for missing values were counter-intuitive (e.g. a higher weight for missing than agreement on day of birth). This was due to small m- and u-probabilities (<0.01) for missing values combined with the log-likelihood scaling convention.

**Table 1:** Initial weight estimates based on first training dataset (records agreeing on NHS number or hospital number).

	**Match weight**		
	**Agreement between identifiers**	**Disagreement between identifiers**	**One or both identifiers missing**
**NHS number**	12.58	-7.94	-0.17
**Hospital number**	12.80	-2.23	0.10
**Surname**	6.20	-3.88	0.26
**Soundex**	5.26	-3.78	-0.46
**First name**	5.19	-3.22	0.25
**Day of birth**	1.28	-6.08	1.66
**Month of birth**	1.18	-6.81	1.66
**Year of birth**	0.91	-6.68	1.66
**Sex**	0.92	-5.63	-0.39

To deal with these problems, record pairs that included completed NHS number, hospital number, first name, surname, postcode and date of birth were extracted and a set of deterministic rules applied (Table 2). Pairs agreeing on a deterministic rule were manually reviewed to remove false-matches based on disagreement between other identifiers, which was possible due to the high-level of identifier completeness. This deterministic process identified 6001 links.

**Table 2:** Identification of links through deterministic linkage.

**Deterministic rule**	**Number of agreeing pairs**	**Number of links**	**Number of non-links**
NHS number or hospital number	4595	4586	9
First name, surname and date of birth	832	832	0
Postcode prefix and postcode suffix	538	416	122
Postcode prefix or postcode suffix and date of birth	94	52	42
At least 2 elements of date of birth and either first name or surname	1559	115	1444
Total reviewed	7618	6001	1617

**Figure 6:**
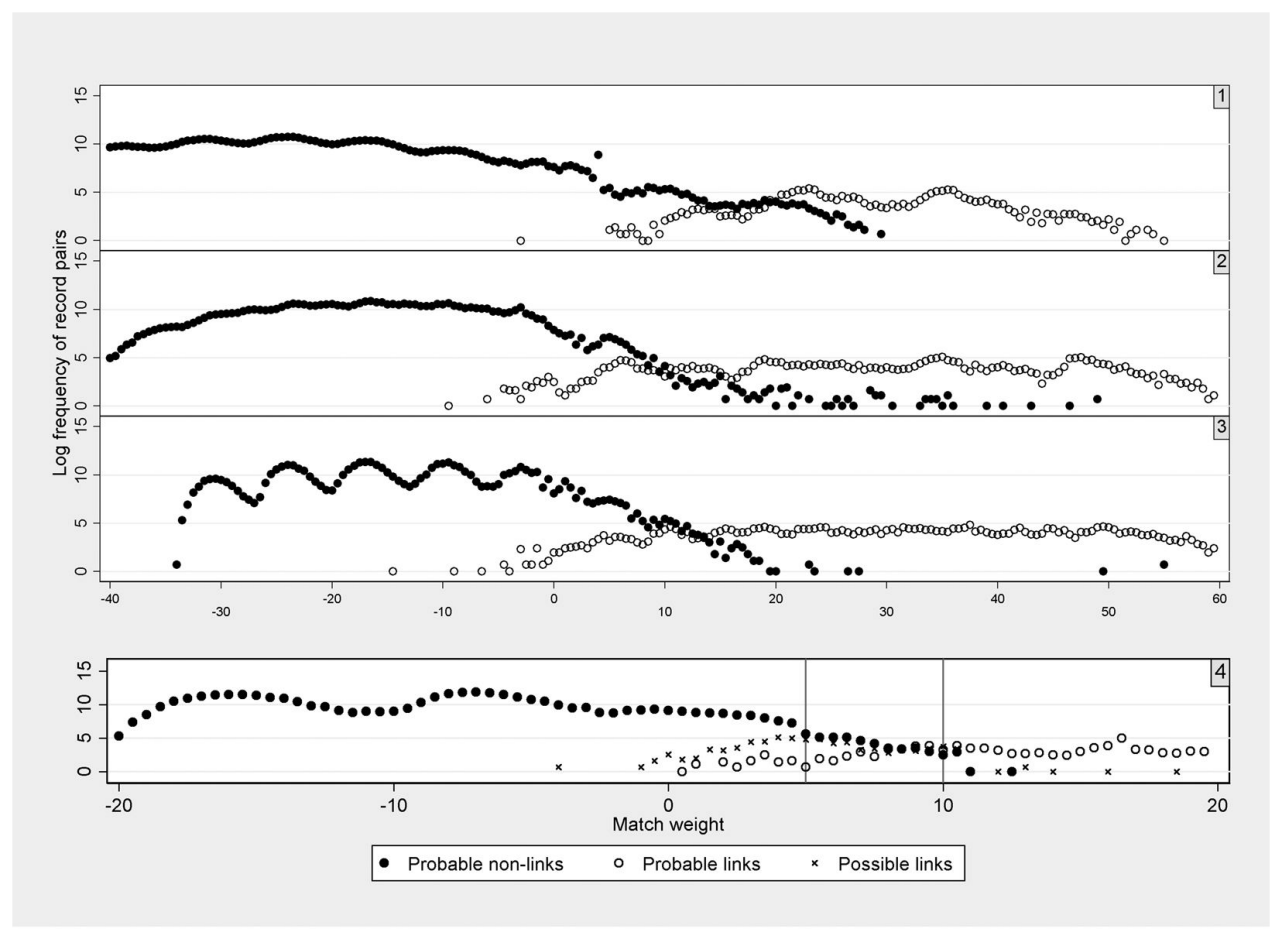
Four iterations for match weight calculation. Lines=thresholds.

Final match weights were then calculated for the remaining records pairs, based on agreement or disagreement on date of birth (combined variable), Soundex, sex and location (Table 3). The relaxed threshold was set at 5, which was chosen to include the majority of probable and possible links whilst not including many non-links (Figure 6). The conservative threshold was set at 10, which was chosen to exclude the majority of non-links, whilst not excluding many probable or possible links.

**Table 3:** Final probabilistic match weights.

	**Match weight**	
	**Agreement**	**Disagreement**
**Soundex**	5.18	-4.05
**Date of birth**	4.66	-6.89
**Sex**	0.91	-4.70
**Location (PICU / lab)**	5.53	-1.06

Calculation of joint match probabilities confirmed that the independence assumption did not hold (Table 4). 

**Table 4:** Match probabilities under independence and dependence assumptions.

**Agreement pattern**				**Assuming dependence**		**Assuming independence**	
**Sex**	**Soundex**	**Dob**	**Location**	**P(NM|g)**	**P(M|g)**	**P(NM|g)**	**P(M|g)**
0	0	0	0	1.000	0.000	1.000	0.000
0	0	0	1	1.000	0.000	1.000	0.000
0	0	1	0	1.000	0.000	1.000	0.000
0	0	1	1	1.000	0.000	0.998	0.002
0	1	0	0	1.000	0.000	1.000	0.000
0	1	0	1	0.993	0.007	0.999	0.001
0	1	1	0	0.883	0.117	0.957	0.043
0	1	1	1	0.600	0.400	0.530	0.470
1	0	0	0	1.000	0.000	0.999	0.001
1	0	0	1	1.000	0.000	1.000	0.000
1	0	1	0	0.998	0.002	0.999	0.001
1	0	1	1	0.949	0.051	0.883	0.117
1	1	0	0	1.000	0.000	1.000	0.000
1	1	0	1	0.985	0.015	0.980	0.020
1	1	1	0	0.175	0.825	<0	>1
1	1	1	1	0.009	0.991	<0	>1

### BSI rate: Evaluation of bias due to linkage error

A total of 6001 (deterministic), 6787 (highest-weighted conservative) and 8490 (highest-weighted relaxed) links were identified. Retaining only the first episode per admission and removing admissions within reporting gaps resulted in 3626, 4651 and 4043 admissions with BSI using each method. Prior-informed imputation identified 4549 admissions with BSI. Compared with gold-standard data, prior-informed imputation provided the least biased estimate of BSI rate (Table 5). 

**Table 5:** Estimated bias based on gold-standard data (BCH and OUH).

**Classification**	**Total links**	**BSI rate**	**% Bias**
*Gold-standard*	*426*	*3.87%*	
Deterministic	125	1.14%	-70.5%
Highest-weighted: Relaxed threshold	492	4.47%	15.5
Highest weighted: Conservative threshold	418	3.80%	-1.9
Prior-informed imputation	424	3.85%	-0.5

After adjusting for -0.5% linkage bias and 80-95% estimated ascertainment, the crude BSI rate increased from 5.79% (initial PII estimate) to 6.13-7.28% (adjusted estimate).

### Representativeness of gold-standard data

BCH and OUH had lower than average BSI rate (2.69% and 4.64% for respective PICUs compared with 5.79% overall). The distribution of identifiers within LabBase2 was also different for these two PICUs. LabBase2 records from BCH were more likely to have completed Soundex, date of birth and sex, but less likely to have completed NHS number, name or postcode. LabBase2 records from OUH were more likely to have completed NHS number and name, but less likely to have completed postcode and date of birth.

## Discussion

Our study demonstrates that linkage of PICU admission data with national BSI surveillance is possible but that results based on these data vary according to the degree of under-ascertainment and bias introduced through linkage of incomplete or imperfect identifiers. We show that reporting gaps and under-ascertainment in national surveillance data lead to under-estimated rates of BSI, but that this can be measured through the use of gold-standard data. We also show that errors occurring during linkage can cause under- or over-estimated rates based on data linked using deterministic linkage only or highest-weight classification, but that prior-informed imputation can provide less biased results. 

Reasons for the under-ascertainment in LabBase2 cited by laboratories are lack of staffing provision, IT system compatibility issues and upload failures. We took a conservative approach to removing data points within periods of incomplete reporting, but this required a certain amount of subjectivity due to the fluctuating nature of BSI reports. PHE is currently developing a new surveillance system that aims to improve data capture. However, this type of data quality issue is relevant to many routinely collected datasets used for health research, and analyses based on these data need to carefully assess how poor data quality might affect results. 

Gold-standard data provide a convenient means for evaluating both ascertainment and bias due to linkage error. This requires the assumption that the gold-standard datasets are representative of the larger dataset of interest. Firstly, we assumed that data capture from RLH and BCH reflected ascertainment in LabBase2 more generally, and that ascertainment was relatively constant over time. However, ascertainment based on these laboratories is likely to be overestimated, as RLH and BCH consistently submit data. Therefore final estimates of BSI rate may be under-estimated. Secondly, we assumed that bias due to linkage error in BCH and OUH was representative. Completeness of identifiers differed between laboratories, and linkage error was therefore distributed non-randomly. Although non-random error can introduce bias into results, prior-informed imputation has been shown to be particularly effective at handling this type of error[12].

This study demonstrates that linkage between routine datasets is complex and requires a number of steps. Firstly, calculation of appropriate match weights requires an iterative process and time-consuming manual review. Calculation of joint match probabilities avoids relying on independence assumptions that often fail, but the most effective ways of estimating such probabilities are still being debated. Current work is investigating this issue. 

Secondly, evaluation of data and linkage quality is required so that potential sources of bias can be identified. Bias due to linkage error can have dramatic effects on analyses based on linked data[13,14,16]. In particular, comparisons of units based on linked data may be biased by differing data quality, and such potential bias needs to be evaluated when using linked data for this purpose. 

Gold-standard data is one way to measure linkage bias, and this was practically possible in our study since linkage and analysis could be performed within the same department (PHE have permission to access patient-identifiable data for the purposes of surveillance). However, this is a special case, as clinical and identifiable data are often separated to protect patient privacy[22]. Appropriate evaluation of linkage success should be presented in reporting analysis based on linked data, to allow meaningful interpretation of results. Careful coordination between linkage and analysis is required so that research based on linked data can be reliable and transparent, whilst data confidentiality is preserved. 
